# Case of leprosy in child

**DOI:** 10.11604/pamj.2022.42.324.35031

**Published:** 2022-08-31

**Authors:** Archana Thaware, Renu Rathi

**Affiliations:** 1 Department of Kaumarbhritya, Mahatma Gandhi Ayurved College Hospital and Research Centre, Datta Meghe Institute of Medical Sciences, Sawangi, Wardha, Maharashtra, India

**Keywords:** Leprosy, *Mycobacterium leprae*, nerve, Schwan cell

## Image in medicine

A 7-year-old male patient was brought to Outpatient Department (OPD) by his parents with complaints of swelling of all fingers with blackish-colored multiple ulcers seen on the fingers and nails. Nail beds were lost on the same fingers as shown in figure and no complaints of pain or tenderness in the cuboidal region. These symptoms were present consistently since six years started at the age of 8 months. In family history, positive contact history confirmed as his uncle had the same complaints. Based on the typical symptoms such as no pain sensation in the fingers, thickness of the nerve at the cuboidal site, multiple ulcerative lesions on and off on the fingers, and positive family history of contact, patient is clinically diagnosed with leprosy. The treatment started as WHO recommended multi-drug therapy (MDT) regimen. It consists of medicines and respective doses of Dapsone (2 mg/kg daily), Rifampicin (10 mg/kg once a month) and Clofazimine (50 mg twice weekly) for 6 months continuously along with wound care, eye care, taking proper treatment and maintaining hygiene and prevention of more wounds by advising to use gloves, socks, and shoes. We suggested consuming enough nutritious food. After 15 days of starting treatment, some symptoms like reduced swelling of fingers and slight better wound healing state observed. The oral medication was continued for the next six months to prevent a relapse of the disease. Initially few follow-up were taken for every 15 days till two months, then monthly for four months and observed the remission. The final diagnosis was leprosy, while the differentials included Leash Nyhan syndrome, Proteus syndrome and Systemic sclerosis.

**Figure 1:**
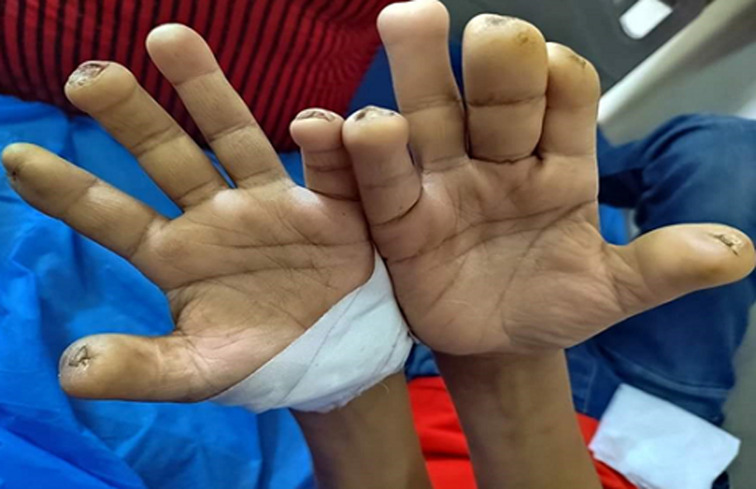
leprosy

